# Analysis of personality traits’ correlation to facial width-to-height ratio (fWHR) and mandibular line angle based on 16 personality factor in Chinese college students

**DOI:** 10.1371/journal.pone.0278201

**Published:** 2022-12-07

**Authors:** Hongpeng Lv, Tianfang Wang, Jing Zhang, Zhaolan Liu, Jian Dong, Haotian Xie, Yingying Yang, Peiwen Xue, Yuwen Che, Pengpeng Han

**Affiliations:** 1 School of Chinese Medicine, Beijing University of Chinese Medicine, Beijing, P. R. China; 2 Department of Student Affairs, Beijing University of Chinese Medicine, Beijing, P. R. China; 3 School of Humanities, Beijing University of Chinese Medicine, Beijing, P. R. China; Universiti Sains Malaysia, MALAYSIA

## Abstract

Facial appearance reveals clues about personality. Studies have found that facial width-to-height ratio (fWHR) correlates with some personality traits, and mandibular morphology as a potential facial feature that might have correlation with personality traits. Therefore, a face recognition study was carried out to explore the personality traits’ correlation to both fWHR and bilateral mandibular line angles. Specifically, face images of 904 college students in China were collected and measured, with the personality traits evaluated using the 16 Personality Factor Questionnaire. Analyses revealed that the average bilateral mandibular line angle of the male were significantly more extensive than that of the female, while the fWHR of the female was significantly more extensive than that of the male. We found facial features (fWHR and average bilateral mandibular line angle) were correlated with 16PF in the canonical correlation analysis and the loadings of bilateral mandibular line angles were greater than that of fWHR. The fWHR was significantly negatively correlated with the scores of sensitivity and self-reliance in male but none of the factors related to fWHR in female. The bilateral mandibular line angles were significantly negatively correlated with the scores of social boldness in male, and were significantly negatively correlated with the scores of vigilance and apprehension in female. Over all, the correlations between fWHR, average bilateral mandibular line angle and certain 16PF factors in male and female tend to be different, suggesting that such correlations might vary with gender. In the future, mandibular morphology could be selected as a potential indicator in facial perception. The limitations of this study were the participants were limited to 18–30 years of age and the mandibular morphology was not measured with anthropometry, which could be further improved in future studies.

## Introduction

The face plays a crucial role in human interaction. Through observation of facial features, one can not only speculate on basic information of a person such as age and gender, but also make subjective judgments of an individual’s health, attractiveness, and personality, etc. [1–4]. Personality is a relatively stable and lasting trait, tendency, or pattern of an individual [5]. It affects an individual’s interpersonal interaction [6], emotion [7], and health status [8, 9], etc. Studies have found that traits of personality can be effectively inferred by analyzing static neutral facial expression images [10–12]. In the present, more and more researches have explored such a relationship and confirmed that there exists intrinsic connection between biological specific facial features and personality in human or even other species [13, 14]. Previous reports have found facial symmetry is significant associated with the Big Five Personality factors [15]. The most widely studied indicator is the facial width-to-height ratio (fWHR), which is found to have a positive correlation with various personality traits, including achievement drive [16], unethical behavior [17], perceived dominance [18], aggression [19, 20], and risk-taking [21].

**Fig 1:**
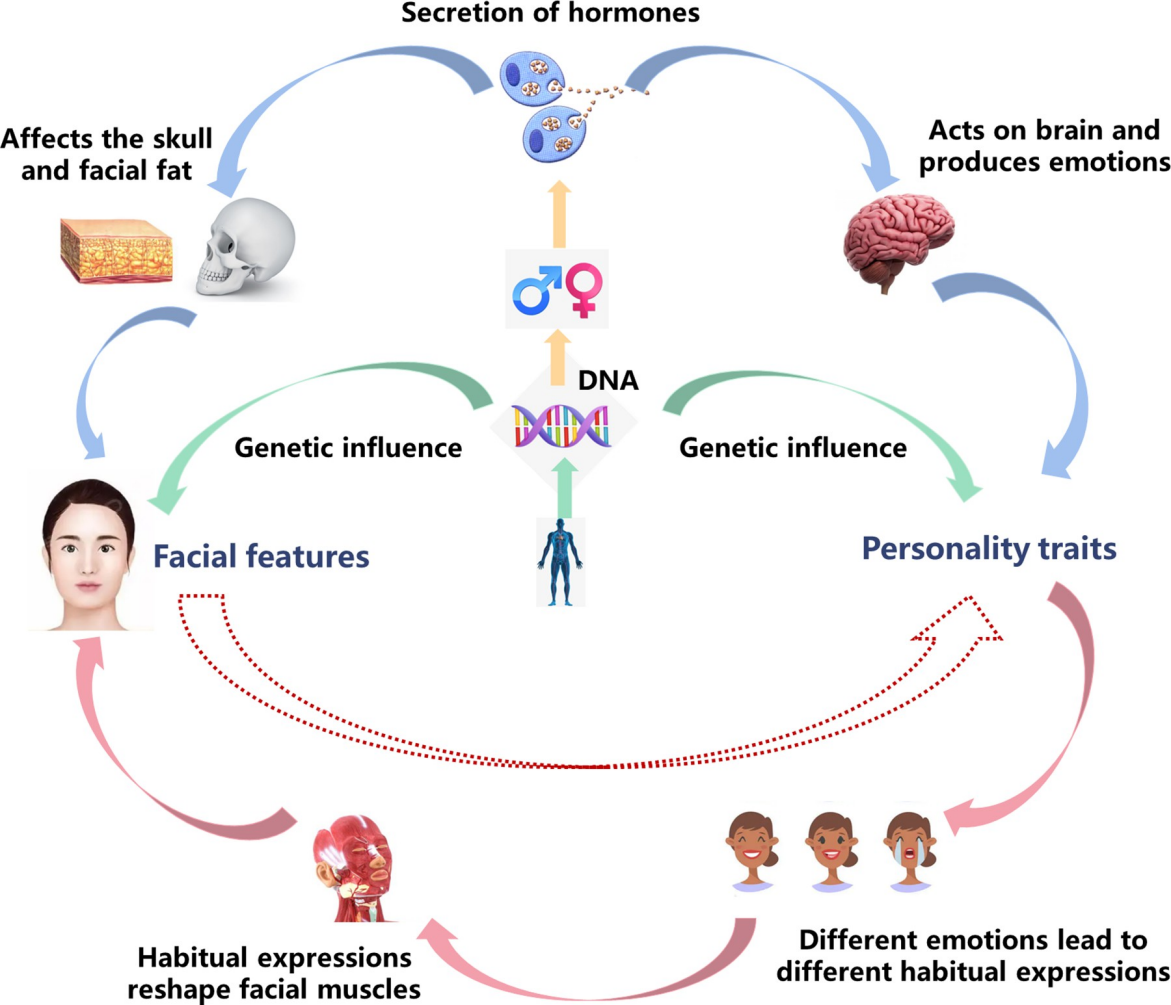
Theoretical support for the correlation between facial features and personality traits. ***Note***: The *green* arrow represents the effects of genetic influence, the *yellow* arrow represents the effects of gender dimorphism, the *blue* arrow represents the neuroendocrine basis, and the *pink* arrow represents the habitual expressions that reshape facial muscles.

As researchers have found coincidental correlations between facial features and personality traits, many studies began to explore their underlying correlation. There is theoretical support for the correlation between facial features and personality traits (Fig 1). Firstly, from a physiological point of view, although facial features and personality development can be affected by environment, they are also closely related to genetic factors. Roosenboom J [22] has found that rs12150660 and rs1799941 genes could affect the morphology of the mandible. What’s more, Terracciano A [23] has found a number of Big Five Personality factors are association with genes, for example, neuroticism with SNAP25 (rs362584), extraversion with BDNF and two cadherin genes (CDH13 and CDH23). Secondly, personality traits are related to the behavioral pattern of emotional expression habit. Habitual emotional expression may shape the static features of the face, leading to the formation of wrinkles and the change of facial muscle outline [24]. Besides, according to neuroendocrine theory, previous studies have proved that different types and levels of hormone secretion can influence skull growth [25–27]. For example, it has been found that facial width-to-height ratio and mandibular morphology are associated with testosterone secretion [22, 28]. Different hormones are found to drive some specific personality traits too, specifically, testosterone and cortisol can effectively regulate individual aggressive and dominant sexual behavior [29, 30]; while on the other hand, estrogen shows the opposite effect on individual’s aggressive behavior [31]. What’s more, many researchers have found that there is a close relationship between the levels of sex hormone secretion and personality in different genders, which results in gender dimorphism. Takahashi K has found the level of aromatase (an enzyme that converts androgen into estrogen in the amygdala) in male’s brain is associated with harm avoidance, persistence, and self-transcendence; while in contrast, aromatase level in female’s brain has been associated with personality traits such as aggression, novelty seeking and self-transcendence [32].

According to previous studies, we have found that some facial morphological features are relatively correlated with personality traits. In the review, we found that the mandibular morphology is genetically heterogeneous, also related to endocranium, which just like fWHR, considered to be a facial feature of sexual dimorphism. However, at present there is seldom research to explore the correlation between mandibular morphology and personality traits. Since this was designed as an exploratory study, we intended to explore the possible correlations between fWHR or mandibular morphology and personality traits, and to provide evidence for relevant explorations in future.

## Methods

### Participants

There were 904 participants (226 males, 678 females), which were all students of Beijing University of Chinese Medicine recruited through an on-campus advertisement from November 21, 2020 to January 9, 2021. The study was carried out in the language of Chinese.

Inclusion criteria: students of Beijing University of Chinese Medicine (undergraduates, master and Ph.D. candidates), Han nationality, 18–35 years old; with no noticeable morphological changes in facial appearance; voluntarily participated in this study, and signed the informed consent form; without apparent physical or mental illness, or physical discomfort (such as severe cold, headache, dysmenorrhea, depression, anxiety, etc.) that prevent them from completing the questionnaire; had not participated in any relevant questionnaire in the past month.

Exclusion criteria: those who had undergone plastic surgery on the face or had not completed the questionnaire, or those whose physical discomfort on the day of the questionnaire evaluation would lead to biased results.

### Ethical approval

This cross-sectional study was approved by the Medical and Experimental Animal Ethics Committee of Beijing University of Chinese Medicine (Ethics batch number: 2020BZYLL0610). The individual in this manuscript had given written informed consent (as outlined in PLOS consent form) to publish these case details.

### Image acquisition

The place of data collection and investigation in this study was the Laboratory in the TCM Diagnostics Department of Beijing University of Chinese Medicine. The room temperature of the laboratory was kept at (24 ±2°C). According to the international lighting industry standard, the display index was set as Ra:90 with color temperature 5000-6000K for good background absorbency by using of light avoidance studio combined with 20w light.

The subjects were instructed to sit on a swivel chair with adjustable seat height and fixed distance from the camera, keep their upper body upright to let the nose tip stay at the same level as the lens, and keep their lower jaw slightly lowered. The subjects’ two-dimensional images of the face on the frontal plane were collected to obtain clear and complete facial information. The subjects should display a neutral facial expression, without apparent angle deflection of the head or makeup at the time of collection. Glasses or other items covering the face should be removed to thoroughly expose the forehead, ears, and neck.

### Measurement of facial features

**Fig 2:**
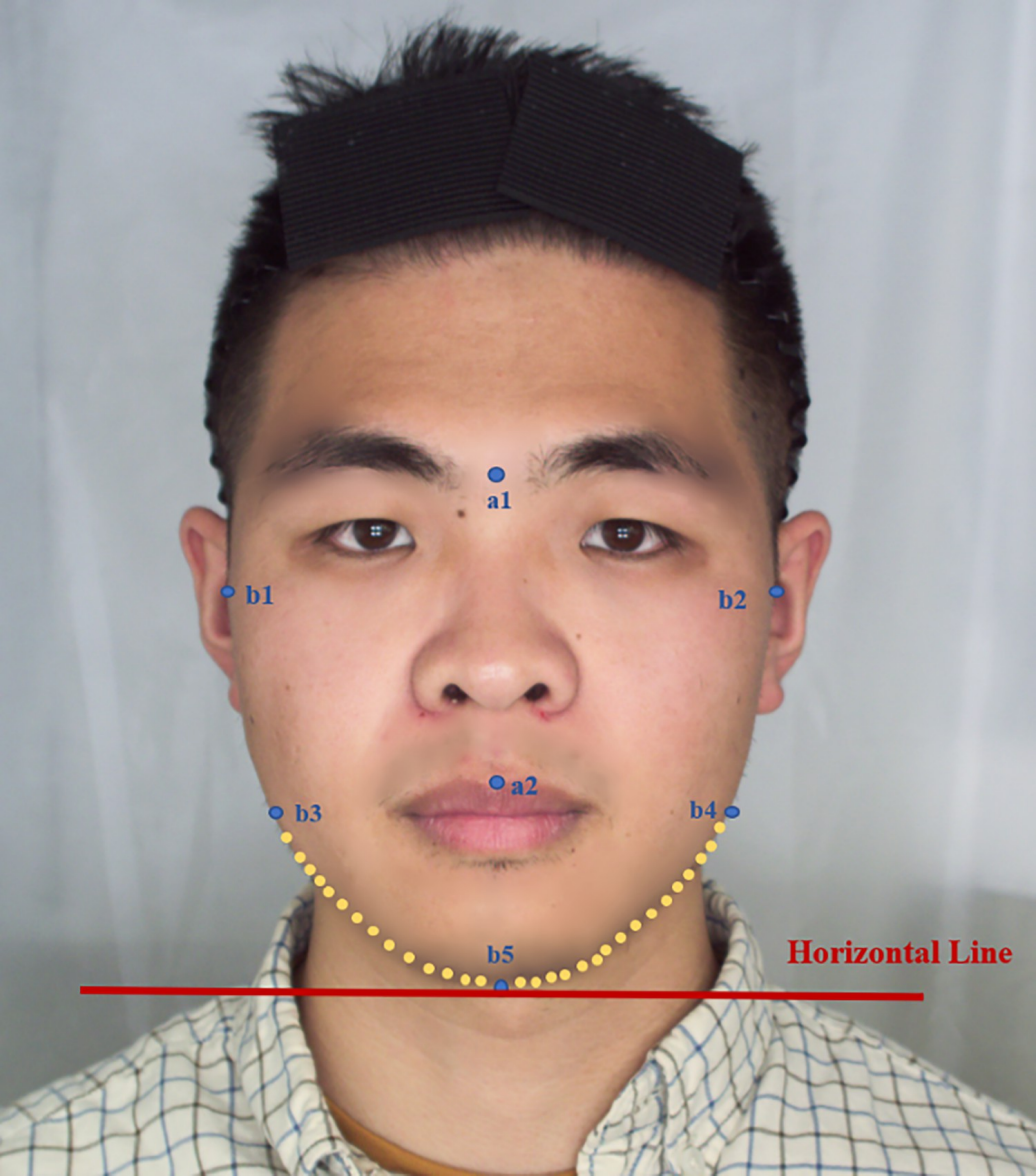
Location of identification indicators related to facial features based on objective measurement data. fWHR = (b1-b2)/(a1-a2)×100; mandibular line angle: the angle between the fitted line and the horizontal line formed by 50 yellow points; blue point: identification indicator; yellow point: indicator for bilateral mandibular contour recognition; red line: horizontal line.

The facial features involved in this study were as follows: Ⅰ) fWHR: the ratio of face breadth to facial height. Face breadth referred to the distance between the right and left zygions (b1-b2); and facial height was the distance between the lip peak (a2) and the brows (a1). Ⅱ) Bilateral mandibular line angle: 50 points of each mandible were taken to fit a line on each side, and the mandibular line angle referred to the one between the fitted line and the horizontal lines. This facial feature can show the degree of square and narrow of the jaw; for example, the larger the mandibular line angle is, the narrower the jaw would be. The specific measurement was positioned as shown (Fig 2). The FaceDiag software developed by the Institute of Microelectronics of the Chinese Academy of Sciences was used to process the images, identify the above location points and measure the feature proportion automatically.

### Assessment of personality

A modified Chinese version of the 16 PF questionnaire for adults (with 187 measurement items) was applied in this study. Each subject was required to complete the 16 PF questionnaire via mobile phone in the Psychological Education Information Management System. The evaluation results were downloaded from the backend server. The above factors were assessed on a scale of 1–10 (for example, for the factor of warmth, 1 for reserved, and 10 for warm). The personality factors tested by 16PF were as follows:

- Warmth (A), reserved/warm
- Reasoning (B), concrete thinking/abstract thinking
- Emotional stability (C), reactive/emotionally stable
- Dominance (E), submissive/dominant
- Liveliness (F), serious/lively
- Rule consciousness (G), expedient/rule conscious
- Social boldness (H), shy/bold
- Sensitivity (I), unsentimental/sensitive
- Vigilance (L), trusting/vigilant
- Abstractedness (M), practical/abstracted
- Privateness (N), forthright/shrewd
- Apprehension (O), self-assured/apprehensive
- Openness to change (Q1), traditional (conservative)/open-to-change
- Self-reliance (Q2), group-dependent/self-reliant
- Perfectionism (Q3), tolerates disorder/perfectionistic
- Tension (Q4), relaxed/tense

### Statistical analyses

Considering multicollinearity in the left and right mandibular line angles, the average values of bilateral mandibular line angle were used to conduct statistical analysis. T-test was used to compare the means of fWHR and average bilateral mandibular line angle between male and female. Canonical correlation analysis, a multivariate statistical analysis method, was carried out to explore the overall linear correlation between facial features (fWHR and average bilateral mandibular line angle) and personality traits (16PF factors). Multiple linear regression was used to analyze the separate correlations between fWHR or average bilateral mandibular line angle and 16PF factor scores, as well as 8 factor scores of second-order calculations. All statistical analyses were performed with IBM SPSS Statistics for Windows (Version 25.0. Armonk, NY: IBM Corp.). The level of statistical significance was set at *P* = 0.05.

## Results

### Subject basic information

This study recruited 904 college students who met the inclusion criteria for this study, with an average age of 21.25 ± 3.22 years old. Among them, 226 cases were male (25.00%), with the average age of 21.59 ±3.45 years old, and 678 cases were female (75.00%), with the average age of 21.16 ± 3.45 years old.

### Comparison of fWHR and average bilateral mandibular line angle between male and female

As shown in Table 1, there were differences in fWHR and average bilateral mandibular line angle between male and female. Specifically, the angle of the bilateral mandibular line in male was significantly higher than that in female (*P<0*.*01*), indicating that females may have a relatively wider jaw. The fWHR in female was significantly higher than that in male (*P<0*.*01*), showing that female seemed to have a relatively wider face.

**Table 1:** Comparison of fWHR and average bilateral mandibular line angle means between male and female with T tests.

Facial features	Gender	N	Mean	SD	*t*	*P*-values
fWHR	Male	226	1.888	0.121	-4.095	<0.001
	Female	678	1.925	0.110		
average bilateral mandibular line angle	Male	226	38.997	1.937	7.530	<0.001
	Female	678	37.872	1.969		

### 16PF factors’ correlation to fWHR and bilateral mandibular line angle in both genders

The canonical correlation coefficients of males and females are presented in Table 2. Only the first canonical function’s correlation was statistically significant (*P* < 0.05, F-test) both in male and female.

**Table 2:** 16PF factors (psychological feature, *A-Q4*)’s correlation to fWHR and average bilateral mandibular line angle (facial morphology feature).

Gender	Pair of canonical variables	*r*	*F*	*df*	*P* values
**Male**	1	0.488	2.602	32.000	<0.001
	2	0.297	1.351	15.000	0.174
**Female**	1	0.312	2.541	32.000	<0.001
	2	0.131	0.775	15.000	0.706

The loadings and cross loadings of the variables for the 1^st^ canonical function were shown in Table 3. For the loadings of the variables for function 1, we found the absolute values of loadings and cross loadings of bilateral mandibular line angle were larger than fWHR both in male and female. Loading of bilateral mandibular line angles was positively correlated with 16PF in male but negatively correlated in female. The most important predictors of 16PF were social boldness (loading: -0.667) in male and apprehension (loading: 0.694) in female.

**Table 3:** Canonical weights, loadings and cross-loadings for the 1^st^ composite scores of the indicators of facial features and 16PF factors (*A-Q4*) from 904 volunteers.

Variables	Male		Female	
	Loadings	Cross loadings	Loadings	Cross loadings
**Independent variables**				
fWHR	0.033	0.016	-0.303	-0.094
average bilateral mandibular line angle	0.946	0.462	0.998	0.311
**Dependent variables**				
Warmth (A)	-0.171	-0.084	-0.155	-0.048
Reasoning (B)	-0.203	-0.099	0.158	0.049
Emotional stability (C)	-0.081	-0.039	-0.004	-0.001
Dominance (D)	-0.115	-0.056	0.039	0.012
Liveliness (F)	-0.097	-0.047	-0.093	-0.029
Rule consciousness (G)	0.055	0.027	0.067	0.021
Social boldness (H)	-0.667	-0.326	0.133	0.042
Sensitivity (I)	-0.144	-0.070	0.025	0.008
Vigilance (L)	0.058	0.028	-0.542	-0.169
Abstractedness (M)	-0.093	-0.045	0.102	0.032
Privateness (N)	0.144	0.070	-0.224	-0.070
Apprehension (O)	-0.145	-0.071	-0.694	-0.216
Openness to change (Q1)	0.083	0.040	-0.056	-0.017
Self-reliance (Q2)	-0.215	-0.105	-0.049	-0.015
Perfectionism (Q3)	0.076	0.037	-0.028	-0.009
Tension (Q4)	-0.143	-0.070	-0.151	-0.047

In order to explore the relationship between facial features and each 16PF factor, we constructed linear regression models in Table 4. In male, we found a significant negative correlation between social boldness and bilateral mandibular line angle (*P* < 0.001, corrected by sequential Bonferroni method), and a negative correlation between sensitivity and self-reliance and fWHR (*P* < 0.05). In female, apprehension and vigilance were significantly negatively correlated with bilateral mandibular line angle (*P* < 0.001, corrected by sequential Bonferroni method).

**Table 4:** Regression analysis of influence of facial feature indicators on 16PF factors.

Gender	Dependent variable	Independent variable	*β*	SE	*β’*	*t*	*P*	95% CI		tolerance	VIF
**Male**	**Social boldness (H)**	(constant)	15.764	3.604		4.375	0.000	8.662	22.865		
		age	0.082	0.036	0.141	2.252	0.025	0.010	0.154	0.976	1.025
		fWHR	0.176	1.009	0.011	0.174	0.862	-1.813	2.165	0.905	1.105
		average bilateral mandibular line angle	-0.322	0.063	-0.333	-5.105	**<0.001**	-0.446	-0.198	0.907	1.102
	**Sensitivity (I)**	(constant)	15.525	4.049		3.835	0.000	7.547	23.504		
		age	-0.079	0.041	-0.129	-1.929	0.055	-0.160	0.002	0.976	1.025
		fWHR	-2.287	1.134	-0.140	-2.017	0.045	-4.522	-0.053	0.905	1.105
		average bilateral mandibular line angle	-0.082	0.071	-0.080	-1.162	0.247	-0.222	0.057	0.907	1.102
	**Self-reliance (Q2)**	(constant)	12.940	3.329		3.887	0.000	6.380	19.500		
		age	0.001	0.034	0.002	0.029	0.977	-0.065	0.067	0.976	1.025
		fWHR	-2.368	0.932	-0.176	-2.540	0.012	-4.205	-0.531	0.905	1.105
		average bilateral mandibular line angle	-0.088	0.058	-0.105	-1.507	0.133	-0.203	0.027	0.907	1.102
**Female**	**Vigilance (L)**	(constant)	12.511	1.946		6.428	0.000	8.689	16.332		
		age	-0.032	0.021	-0.059	-1.553	0.121	-0.073	0.008	0.998	1.002
		fWHR	-0.506	0.596	-0.033	-0.850	0.396	-1.676	0.663	0.939	1.065
		average bilateral mandibular line angle	-0.155	0.033	-0.181	-4.636	**<0.001**	-0.220	-0.089	0.940	1.064
	**Apprehension (O)**	(constant)	13.524	2.107		6.420	0.000	9.388	17.660		
		age	0.010	0.022	0.016	0.435	0.664	-0.034	0.054	0.998	1.002
		fWHR	-0.157	0.645	-0.009	-0.244	0.807	-1.423	1.108	0.939	1.065
		average bilateral mandibular line angle	-0.204	0.036	-0.219	-5.650	**<0.001**	-0.275	-0.133	0.940	1.064

***Note***: Bold indicates the correlation is significant at alpha level corrected by sequential Bonferroni method. Each factor of 16PF was the dependent variable, while fWHR, average bilateral mandibular line angle and age were the independent variables.

## Discussion

### The difference between fWHR and mandibular morphology in different genders

In our study, the results show that the fWHR in women is higher than that of men, indicating that women’s faces are wider than men’s (Table 1), and Ozener’s study shows a similar finding [33]. Wen [34] also has concluded similarly to our finding (the subjects are Chinese). Both studies support the results of our study. However, contrary to that, there exist some current studies finding a quite opposite result [19, 35], and some researchers have come to a different conclusion that there is no significant difference in fWHR between sexes in the sample of college students in Ankara, Turkey [33] and even in German [36]. In terms of the mandibular morphology, it is considered to be a sign of dimorphism [37]. Some studies have found that the mandibular angle is larger in male in Chinese Han population than that of females [38], which is similar to our results. Therefore, the fWHR in men and women may be affected by physiological factors like genes and race, external living environment such as living areas and environment, as well as the quality of life [39].

### Personality’s correlation to both fWHR and mandibular morphology

In our study, the results of canonical correlation analysis showed that the facial features represented by fWHR and average bilateral mandibular line angle were correlated with the personality traits represented by 16PF factors both in males and females. Recently, more and more studies have found a close relationship between facial features and personality traits [15, 20, 40]; however, previous studies have seldom used 16PF to explore the connection. The results in our studies suggest that facial features do have a certain correlation with 16PF factors, so the results of canonical correlation analysis have confirmed the correlation between facial features and 16PF personality factors.

In order to explore the correlation between fWHR or average bilateral mandibular line angle and each factor of 16PF, we have used multiple linear regression analysis. Considering that familywise errors were likely to occur in multiple linear regression analysis, we used Bonferroni correction. Even though no significant result has been obtained with Bonferroni correction in this study, we are in an effort to show the possible correlation with the following considerations. Firstly, as this study is an exploratory study, it preliminarily explores the correlation between fWHR or mandibular morphology and 16PF factors. Secondly, each dimension of 16PF personality factors is relatively independent and has little correlation with each other. In addition, some researchers have different opinions on multiple tests that this method might neglect to any possible associations [41].

According to the results of Bonferroni correction (Table 4), we’ve found that the correlation between fWHR and personality traits only exists in males, which is consistent with the finding of a previous study that fWHR is associated with personality traits only in males [42]. Previous studies have found that fWHR is a sex hormones regulated facial feature, some studies have explored the relationship between fWHR and testosterone, to try to explain the significant correlation between fWHR and personality characteristics. Noser E. has identified that men with a wider fWHR generally has higher level of testosterone [28], which has been considered effective in regulating individual aggression and dominance [29, 30, 43]. However, other studies have found the opposite result, which have found no significant association between fWHR and testosterone and testosterone-linked traits in adolescent or adults [44, 45]. Therefore, the reasons for the correlation between fWHR and personality traits may be complex and not solely related to sex hormones. Besides, we’ve found a negative correlation between fWHR and factors of 16PF including sensitivity and self-reliance in males. In previous studies, male’s fWHR has been found to be positively correlated with career success [46], sense of power [17], and dominance [18]. We interpret such divergences as a result of complicated factors such as different sample sizes, race, quality of education, economic privilege, age and so on.

In addition, we’ve found a potential relationship between mandibular morphology and personality traits. The average bilateral mandibular line angle seems to be negatively correlated with social blindness in male, and negatively correlated with vigilance and apprehension in female, which has been rarely reported in previous studies. Cranium growth and development are affected by many factors, including genes, hormones, nutrients, and epigenetic factors, which interact to form a specific shape of a craniofacial bone. Any interference in this mechanism may lead to changes in the morphology of the craniofacial bone [47]. Based on that, the correlation between mandibular line angle and personality traits in our study can be explained by taking into consideration the effect of certain hormones on personality traits. Firstly, the formation of mandibular morphology is related to testosterone, Roosenboom J. have found that mandibular morphology is also closely related to testosterone levels, as well as genes involved in regulating testosterone secretion [22]. Accordingly, men with prominent mandibular are perceived as being more masculine [48, 49]. While the level of testosterone secretion is related to personality formation. In other previous researches, it is found that increasing the level of testosterone in healthy men from regular to high range (all within the normal range) can increase the risk-taking behavior [50, 51]. All of these studies support the correlation between high testosterone level and behaviors as the result of a bold personality, which helps us to speculate that the mandibular line angle may be related to the secretion level of testosterone. Secondly, according to the previous studies, mandibular morphology is also related to growth hormones, it has been found that children (especially girls) with growth hormone deficiency have smaller mandibular ramus height, such abnormal development of the mandible can be improved after growth hormone treatment, indicating that the formation of mandibular morphology is related to growth hormone, and it is more significant in female [52]. What’s more, researches on patients with acromegaly, which is caused by excessive secretion of growth hormone because of anterior pituitary hormone-active adenomas, show that such patients are more likely to develop negative emotions, such as worry, sadness, and anxiety [53, 54]. In our study, we’ve found that there was a negative correlation between average bilateral mandibular line angle and vigilance or apprehension in female. Combining our results and the above research findings, we speculate that the correlation between female mandibular morphology and personality may be related to the level of growth hormone secretion.

The correlation between facial features and personality traits can not only be explained by neuro-endocrine theory, but also proposed by facultative personality calibration, which means that the way of cultivating personality can adapt to our innate genetic performance to the maximum extent, such as our body size, strength and facial morphology [55]. People with higher levels of appearance attractiveness are also more likely to develop an extroverted personality [56]. Aaron Lukaszewsk [57] has found that men who are stronger (this does not apply to women) and more physically attractive are more likely to be willing to have sex with women who have no intimate relationship. Therefore, due to the influence of social relations, facial morphology itself also affects the development of personality.

Moreover, our study has used canonical correlation analysis and found the loadings and cross loadings of the variables of average bilateral mandibular line angle seems to be much larger than fWHR in both male and female(Table 3). Numerous previous studies have supported the correlation between fWHR and personality traits especially in male [21, 46], although some studies have stated that there is no correlation between fWHR and personality [58]. The results of our study shows that bilateral mandibular line angles might be another worthy indicator to explore in facial perception. And the result of multivariate linear regression analysis in this study has also confirmed the above inference that the average bilateral mandibular line angle is significantly related to 16PF factors (corrected by sequential Bonferroni method both in males and females), while on the contrary, the correlation between fWHR and the 16PF factors is not so significant. This finding indicates that bilateral mandibular line angle is also an important indicator, but the same mandibular morphology in male and female may predict different personality traits. For example, average bilateral mandibular line angle is probably negatively correlated with social boldness in male, but negatively correlated with vigilance and apprehension in female. Therefore, further studies on the correlation between mandibular morphology and personality might also be analyzed with gender considered. As an exploratory study with a limited group sample, we hope to provide some reference for future studies.

In the image acquisition, we have used an industrial camera instead of an ordinary camera for the following two reasons: ① A shorter shutter time. The MARS industrial camera we’ve used is with a frame rate of 32.1fps, which is ten times more than that of a standard camera, so it is convenient to take pictures of non-absolute static objects like a human; ② More accurate data. The naked data output of an industrial camara is suitable for high-quality image processing algorithm, which provides a guarantee for us to obtain more accurate facial features data. Moreover, we control the display index as Ra:90 with color temperature 5000-6000K, for the light source of each participants’ face image was basically the same by using fixed light to avoid being too bright or too dark to affect the image effect. And it is considered that if the color temperature difference is too large, it is possible to identify the skin rash or nevus on the face of some subjects as hairline or other contour margin of face in measurement of facial features.

### Strengths and limitations

In terms of methods, firstly we’ve adopted the 16PF scale to provide richer results regarding personality traits [59] compared with previous studies in which routinely the Big five Personality scale were used [21, 24, 60], thus this study has provided more evidence for exploring the correlation between facial features and personality traits. What’s more, in our study we’ve included students in the same medical college to exclude the influence of different learning environments on personality traits.

Of course, our study also has some limitations. First of all, the sample of this study is only limited to the fixed age group of 18–30 years old students in a certain university, and the sample size of males and females is imbalanced, so further studies can expand and balance the sample size. Secondly, although we paid attention when subjects’ faces were taking frontal images, it was inevitable to cause errors, so we could not distinguish between the left and right mandible line angles caused by the error in taking photos or the facial asymmetry of the subjects themselves. Besides, because the mandibular ramus height and mandibular angle cannot be measured based on a picture of the face, we use recognition technology to measure them so as to form a description of the mandibular morphology. In future studies, each index of mandibular shape can be physically measured in real people when the actual situation allows. What’s more, the results of this study could be helpful to expand the research on the correlation between facial morphological traits and personality, additional studies are needed to verify whether the correlation between mandibular morphology and personality traits is related to neuroendocrine basis.

## Conclusion

In our study, we find there are potentially significant differences in fWHR and bilateral mandibular line angles between males and females, and the correlations between fWHR, average bilateral mandibular line angle and certain 16PF factors in male and female tend to be different, suggesting that such correlations might vary with gender. The correlation between average bilateral mandibular line angle and personality is higher than that between fWHR and personality, indicating that mandibular morphology might be another potential indicator in facial perception.

## Supporting information

S1 Data (XLSX)
